# Anisotropic Growth and Magnetic Properties of α″-Fe_16_N_2_@C Nanocones

**DOI:** 10.3390/nano11040890

**Published:** 2021-03-31

**Authors:** Yong Li, Qifeng Kuang, Xiaoling Men, Shenggang Wang, Da Li, Chuljin Choi, Zhidong Zhang

**Affiliations:** 1 Shenyang National Laboratory for Materials Science, Institute of Metal Research, Chinese Academy of Sciences, 72 Wenhua Road, Shenyang 110016, China; yli14s@163.com (Y.L.); qfkuang18b@imr.ac.cn (Q.K.); xlmen19b@imr.ac.cn (X.M.); sgwang@imr.ac.cn (S.W.); zdzhang@imr.ac.cn (Z.Z.); 2 Korea Institute of Materials Science, 797 Changwondaero, Seongsangu, Changwon 51508, Gyeongnam, Korea

**Keywords:** α″-Fe_16_N_2_, permanent magnetic material, one-dimensional nanocones, core/shell structure, anisotropic growth, chemical solution method

## Abstract

α″-Fe_16_N_2_ nanomaterials with a shape anisotropy for high coercivity performance are of interest in potential applications such as rare-earth-free permanent magnets, which are difficult to synthesize in situ anisotropic growth. Here, we develop a new and facile one-pot microemulsion method with Fe(CO)_5_ as the iron source and tetraethylenepentamine (TEPA) as the N/C source at low synthesis temperatures to fabricate carbon-coated tetragonal α″-Fe_16_N_2_ nanocones. Magnetocrystalline anisotropy energy is suggested as the driving force for the anisotropic growth of α″-Fe_16_N_2_@C nanocones because the easy magnetization direction of tetragonal α″-Fe_16_N_2_ nanocrystals is along the c axis. The α″-Fe_16_N_2_@C nanocones agglomerate to form a fan-like microstructure, in which the thin ends of nanocones direct to its center, due to the magnetostatic energy. The lengths of α″-Fe_16_N_2_@C nanocones are ~200 nm and the diameters vary from ~10 nm on one end to ~40 nm on the other end. Carbon shells with a thickness of 2–3 nm protect α″-Fe_16_N_2_ nanocones from oxidation in air atmosphere. The α″-Fe_16_N_2_@C nanocones synthesized at 433 K show a room-temperature saturation magnetization of 82.6 emu/g and a coercive force of 320 Oe.

## 1. Introduction

Permanent magnets can provide a high efficiency and reliability for renewable energy technologies, such as wind turbines and hybrid electric vehicles [1]. Rare-earth-free permanent magnets, expected to be the next generation of permanent magnetic materials, have been paid much attention due to abundant resources, low cost, large coercivity and high Curie temperatures [2]. Body-centered tetragonal (bct) α″-Fe_16_N_2_, being an ordered nitride, is one of the most promising candidates for future applications of rare-earth-free permanent magnets because of the excellent magnetocrystalline anisotropy (7.8 × 10^5^ J/m^3^) and the highest saturation magnetization reported so far [3,4,5,6,7,8]. In the iron–nitrogen binary system, Jack first reported that the metastable phase α″-Fe_16_N_2_ has a giant magnetic moment [9]. Besides α″-Fe_16_N_2_, ferromagnetic iron nitrides include a hexagonal close packed ε-phase with general formula ε-Fe_3_N_1+x_ showing a wide range of composition range with extreme values of −0.40 < *x* < 0.48, and the face-centered cubic γ′-Fe_4_N. Face-centered cubic γ′-Fe_4_N has a ferromagnetic state below its Curie temperature of about 760 K [10], while ε-Fe_3_N (*x* ≤ 0) in a space group P6_3_22 with all the N atoms occupying octahedral interstices (2c site) exhibits a well-ordered structure, showing enhancements of the Curie temperature (*T_C_*) and room-temperature saturation magnetization (*M_S_*) with decreasing N content, with a record-high *T_C_* (632 K) and *M_S_* (192 emu/g) at *x* = 0.88 [11]. The magnetic moment of iron atoms on the 4d site of α″-Fe_16_N_2_ is 3.0 μ_B_/Fe [12]. The *M_S_* of partially ordered Fe_16_N_2_ thin films increases monotonically with an increasing volume ratio of the α″-Fe_16_N_2_ and the N site ordering parameter related on the special arrangement of Fe_6_N clusters [13]. Anisotropic α″-Fe_16_N_2_ magnets were prepared by starting from pure bulk Fe with urea as the nitrogen source [14]. Because of optimal coercivity performance of single-domain nanoparticles, the lower dimensionality of the enhanced hard magnetic properties of magnetic nanostructures has been attractive in the area of hard magnetic nanomaterials [2,15,16]. Well-controlled nanostructures of hard magnets are a possible future choice for controlling texture and magnetic alignment to support high-density magnetic storage and giant energy density [17].

α″-Fe_16_N_2_ powders and nanoparticles were obtained by mechanical ball milling with a solid nitrogen source of NH_4_NO_3_ [18] and ammonia nitrification of iron nanoparticles after being reduced from Fe-oxide nanoparticles [19], showing a coercivity (*H_C_*) as high as 3.35 kOe. Core-shell α″-Fe_16_N_2_/SiO_2_ [20], α″-Fe_16_N_2_/C [21] and α″-Fe_16_N_2_/Al_2_O_3_ nanoparticles [22] were obtained through various successive procedures starting from the reduction of Fe-oxides, followed by nitridation. The core-shell structure can restrict the growth of α″-Fe_16_N_2_ nanoparticles during the nitridation process, which improves the coercivity performance of α″-Fe_16_N_2_ nanoparticles. However, the *M_S_* values experimentally determined in α″-Fe_16_N_2_ nanoparticles were in the range 160–225 emu/g, smaller than the value (290 emu/g) for α″-Fe_16_N_2_ thin film [9,13,23,24]. In nanowire arrays, the dipole–dipole interaction between wires becomes important [25]. By combination of the shape anisotropy and the magneto-crystalline anisotropy, ferromagnetic materials with a high magnetic anisotropy exhibit a high coercivity [15].

The most common synthesis route for iron nitrides is nitridation of iron films, powders and nanoparticles in an NH_3_ atmosphere at high temperatures (≥573 K) [26,27,28,29]. Such high-temperature nitridation routes inevitably create disordered nitrogen atoms in iron nitrides. The chemical solution method is mild and highly effective to synthesize high-purity magnetic nanocrystals with controlled composition, size and microstructure by varying the reaction conditions [16,30]. Recently, nanoscale and stable ε-Fe_3_N_1+x_ (−0.12 ≤ *x* ≤ −0.01) nanoparticles with highly ordered N atoms were obtained by using iron(II) acetylacetonate and tetraethylenepentamine (TEPA) as Fe and N/C sources under a lower temperature (533 K) [11]. Such a novel chemical synthesis route is favorable for preparation of monophasic ε-Fe_3_N_1+x_ (*x* < 0) nanoparticles that were never synthesized previously because the ε-Fe_3_N_1+x_ (*x* < 0) phases are unstable at a temperature lower than their synthesis temperatures [31,32]. Due to thermal instability at ~523 K, the synthesis of α″-Fe_16_N_2_ with highly ordered N atom occupancies is usually at a nitridation temperature lower than 453 K [33,34,35]. Improvements thereof are expected in the chemical synthesis for α″-Fe_16_N_2_ and its magnetic properties. Herein, carbon-coated one-dimensional (1D) α″-Fe_16_N_2_ nanocones with an average length of 200 nm were synthesized by a new simple low-temperature chemical solution method. Anisotropic growth mechanism, structure and magnetic performance of the α″-Fe_16_N_2_@C nanocones were studied.

## 2. Materials and Methods

### 2.1. Chemicals

Iron pentacarbonyl [Fe(CO)_5_, 98%], 1-octadecene (ODE, 90%) and oleylamine (OLA, 90%) were purchased from Aladdin reagent company (Shanghai, China). Absolute ethanol (99.7%) and tetraethylenepentamine (TEPA, 90%) were purchased from Sinopharm Chemical Reagent Co. Ltd. (Shenyang, China). Prior to synthesis, the original TEPA was heated to 588 K in a nitrogen atmosphere and kept at this temperature for 5 h. Other chemicals were used without further purification.

### 2.2. Synthesis of α″-Fe_16_N_2_@C Nanocones

The solution syntheses were carried out at a reaction temperature range from 393 to 553 K with a varied reaction time of 1 and 6 days. When the reaction temperature and reaction time were less than 433 K and 3 days, both Fe_3_O_4_ and α-Fe were usually found in the products because the nitridation reactions were incomplete. In a typical synthesis of α″-Fe_16_N_2_@C nanocones, 10 mL of the treated TEPA, 30 mL of ODE and 10 mL of OLA were mixed into a 250 mL four-neck flask using a Schlenk line under air-free conditions and magnetically stirred throughout the entire reaction process. Under a mixed Ar/H_2_ (95:5) flow, the mixture was first heated to 383 K. The solution was kept at this temperature for 60 min. to remove low boiling-point solvent and oxygen. Then, the temperature was increased to 473 K at 5 K·min^−1^ and a translucent yellow microemulsion was obtained. At 473 K, a mixed solution consisted of 2 mL of Fe(CO)_5_, 6 mL of ODE and 2 mL of OLA in a syringe which was injected into the microemulsion at a rate of 30 mL/h and maintained at this temperature for 30 min. for the formation of Fe nanocrystals (NCs). Subsequently, the reaction system was cooled to 433 K and maintained at this temperature for 6 days, at which temperature α″-Fe_16_N_2_ was synthesized. At room temperature, the product was precipitated by centrifugation at 3000× *g* rpm for 5 min. The precipitate was rewashed in 20 mL of ethanol three times and dried in a vacuum for further characterization.

### 2.3. Characterization

The size, morphology and microstructure of the α″-Fe_16_N_2_@C nanocones were observed using a Tecnai G2 F20 transmission electron microscope (TEM) (FEI Inc., Hillsboro, OR, USA) at 200 kV. The scanning electron microscopy (SEM) images were obtained with a JSM 6301F field-emission scanning electron microscope (FESEM) system (JEOL Inc., Tokyo, Japan). Powder X-ray diffraction (XRD) was recorded on a D/Max-2400 diffractometer (Rigaku Inc., Tokyo, Japan) equipped with a Cu K_α_ radiation source (λ = 0.154056 nm). The composition and surface information of the nanocones were determined by X-ray photoelectron spectroscopy (XPS) (Thermo Fisher Inc., Waltham, MA, USA). The surface of compacted samples was cleaned by argon–ion bombardment. Both the as-prepared and the surface-cleaned samples were analyzed by the XPS to determine the species covering on the surface of samples. Thermal analysis was performed on an STA6000 thermal analyzer (PerkinElmer Inc., Waltham, MA, USA) under N_2_ flow with heating rate of 10 K·min^−1^ between 300 and 900 K. Room-temperature magnetic hysteresis loop and temperature-dependent magnetization in the rising/cooling processes (at *H* = 1 kOe) were carried out using a vibrating sample magnetometer (VSM) in a physical property measurement system (PPMS) (Quantum Design Inc., San Diego, CA, USA) equipped with a superconducting magnet with a maximum magnetic field of 140 kOe.

## 3. Results

### 3.1. Structural Properties

**Figure 1:**
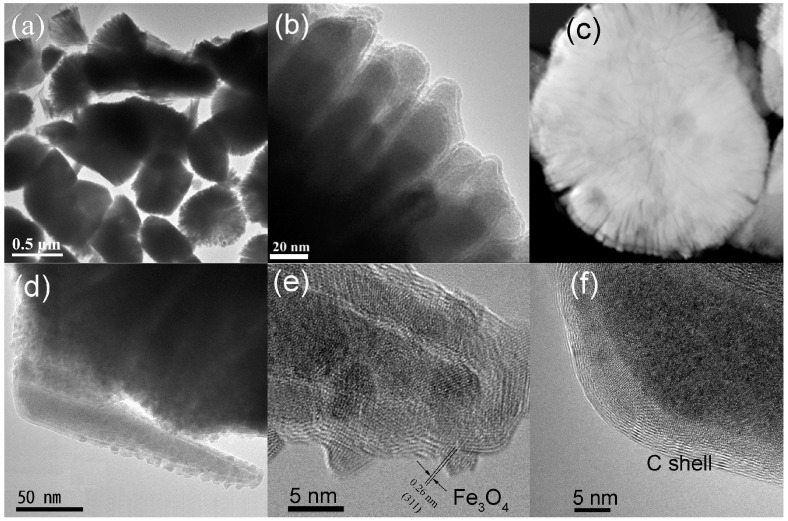
(**a**,**b**) TEM images and (**c**) HADDF image of α″-Fe_16_N_2_@C nanocones synthesized at 433 K for six days. (**d**) TEM image for a separate α″-Fe_16_N_2_@C nanocone and its corresponding HRTEM image for (**e**) Fe_3_O_4_ nanocrystals on the surface of the thinner end and (**f**) carbon shell of the thicker end.

The TEM image in Figure 1a shows that the product synthesized at 433 K for six days consists of fan-shaped particles with a diameter size of about 500 nm, which were formed by an assembly of nanocones (Figure 1b). The microstructure at the edges of the fan-shaped particles shown in Figure 1b and an HADDF image in Figure 1c reveal a relatively flat surface of the fan-shaped particles assembled by two or three layers of nanocones. Such an intricate microstructure forms when the as-synthesized nanocones agglomerate, in order to minimize the magnetostatic energy [29]. The nanocones have different diameters at two ends, which gradually change from ~40 to ~5 nm and the length of the nanocones is about 200 nm (Figure 1d). A small amount of Fe_3_O_4_ nanocrystals with an average particle size of ~4 nm can be observed on the surface of the nanocones. The HRTEM image of a cubic nanocrystal (Figure 1e) shows a lattice fringe with spacing of approximately 0.26 nm, which represents the (311) planes (0.253 nm) of the cubic Fe_3_O_4_ according to the Joint Committee on Powder Diffraction Standards (JCPDS) XRD card (19−0629). The Fe_3_O_4_ nanocrystals should come from the oxidation of excess of Fe nanocrystals (NCs) without carbon coating. The HRTEM image in Figure 1f demonstrates a typical core-shell structure of an individual nanocone with the thickness of the carbon shell being about 2–3 nm. However, there is no evident lattice fringe spacing for the nanocone, indicating the poor crystallization or crystallite constituents with very small grain size.

**Figure 2:**
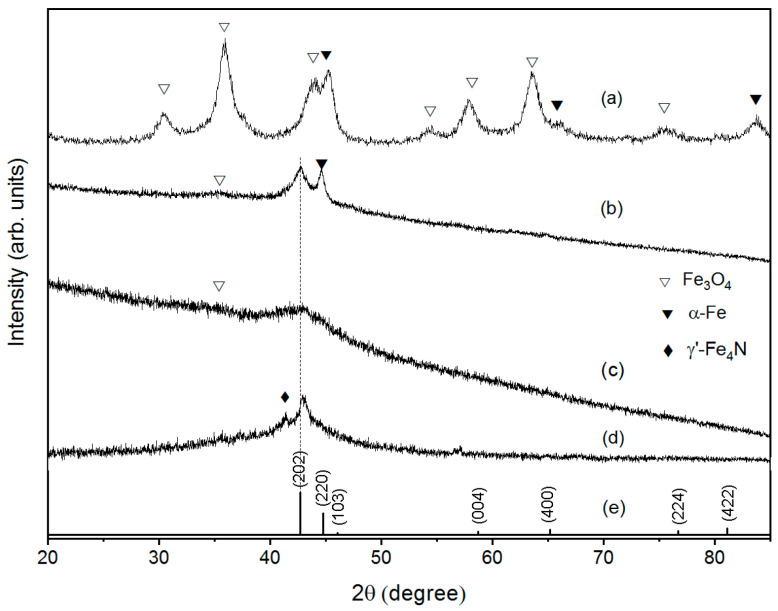
Powder XRD patterns of the products synthesized under different reaction conditions: (**a**) 393 K and three days, (**b**) 403 K and three days, (**c**) 433 K and six days, (**d**) 453 K and six days, (**e**) JCPDS XRD card of #78-1865 for α″-Fe_16_N_2_.

**Figure 3:**
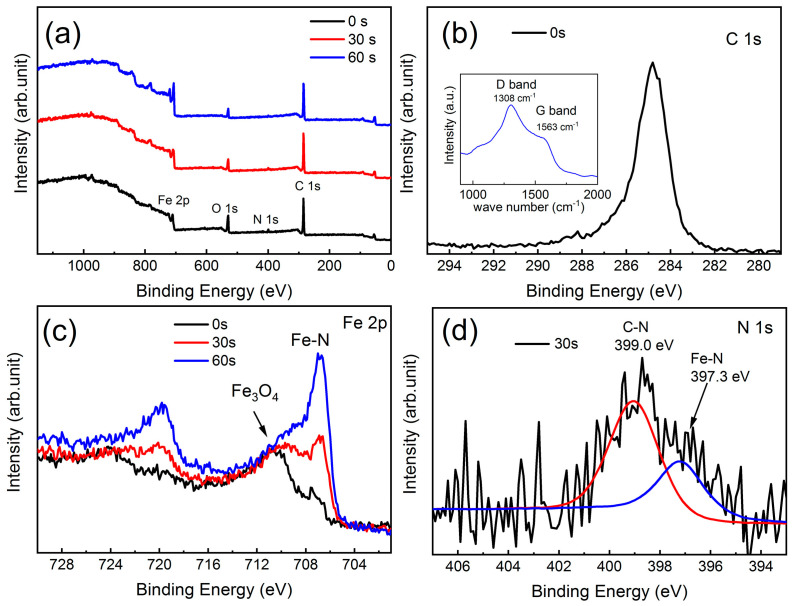
XPS spectra of the α″-Fe_16_N_2_@C nanocones synthesized at 433 K for six days. (**a**) Survey of the sample with the surfaces cleaned for 0 s, 30 s and 60 s, respectively. (**b**) C 1s, (**c**) Fe 2p and (**d**) N 1s spectra. The inset in (**b**) shows the Raman spectrum of the as-prepared α″-Fe_16_N_2_@C nanocones.

Figure 2 shows the phase evolution of final products synthesized at the different reaction conditions, such as the reaction temperature and time. The powder XRD pattern in Figure 2a indicates that the sample synthesized at 393 K for three days consisted of Fe_3_O_4_ and α-Fe. The Fe_3_O_4_ and α-Fe NCs agglomerated into spherical particles under the magnetostatic energy (not shown here). When the nitridation reaction was performed at 403 K for three days, the powder XRD pattern of the as-prepared sample in Figure 2b reveals that the XRD peaks can be indexed to α-Fe with the JCPDS XRD card (06−696), α″-Fe_16_N_2_ with the JCPDS XRD card (78−1865) and a small amount of Fe_3_O_4_ characterized by a weak (311) XRD peak at 2θ = 35.4°. When the reaction temperature and time were increased to 433 K and six days, respectively, Figure 2c shows that the product consisted of the main phase α″-Fe_16_N_2_ and a trace amount of Fe_3_O_4_. There is no α-Fe in the product due to the oxidation of excess of α-Fe NCs without carbon coating, as presented in Figure 1d,e. The XRD peaks of the as-synthesized α″-Fe_16_N_2_ nanocones in Figure 2c match well with the standard XRD pattern of bulk α″-Fe_16_N_2_ (JCPDS card no. 78−1865) that has a tetragonal structure with a space group of I4/mmm (139) and a = b = 5.72 Å, and c = 6.29 Å (Figure 2e). The main peak position of the product with a nanocone shape is consistent with that of (202) for α″-Fe_16_N_2_, indicating that the properly increased reaction temperature and time were helpful to the diffusion of N atoms into α-Fe nanocrystals for the formation of α″-Fe_16_N_2_. However, the (202) XRD peak around 2θ ≈ 42.7° and (220) XRD peak around 2θ ≈ 44.8° for α″-Fe_16_N_2_ emerge as a broad peak, revealing the poor crystallization or α″-Fe_16_N_2_ crystallite with small grain sizes in the nanocones. The average grain size of α″-Fe_16_N_2_ nanocrystals calculated by the Scherrer formula was about 3 nm, in good agreement with the HRTEM result shown in Figure 1d. After raising the reaction temperature and time to 453 K and six days (Figure 2d), the main peak position (2θ) of the product shifted from 42.7° for α″-Fe_16_N_2_ to 43.05° and a new XRD peak at 41.3° emerged for the (111) of γ-Fe_4_N (JCPDS card no. 06−627). This reveals that extra N atoms will transform the α″-Fe_16_N_2_ to a stable phase of γ-Fe_4_N. There was no carbon detected in the XRD patterns due to the slight amount. XPS was used to detect the species covering on the surface of the nanocones. Figure 3a represents the XPS spectra of the as-prepared and surface-cleaned α″-Fe_16_N_2_ nanocones synthesized at 433 K for six days. The binding energies at 284.6 and 285.7 eV in the XPS spectra in Figure 3b reveal ordered and disordered carbon, respectively, on the surfaces of α″-Fe_16_N_2_ nanocones, which is consistent with the Raman data shown as the inset in Figure 3b. Figure 3c shows the XPS spectra of Fe2p. The binding energies of Fe2p_3/2_ at about 706.7 and 710.4 eV should be assigned to α″-Fe_16_N_2_ and Fe_3_O_4_, respectively. The XPS peak area corresponding to Fe_3_O_4_ decreased significantly when we extended the Ar ion sputtering time from 0 s to 60 s, confirming the slight Fe_3_O_4_ existing on the surfaces of α″-Fe_16_N_2_ nanocones. The weak binding energy peak at 397.3 eV featured by the blue line in Figure 3d was assigned to the N element in α″-Fe_16_N_2_, while that at 399.0 eV featured by the red line was assigned to the C-N from TEPA absorbed on the surface of the nanocones.

### 3.2. Anisotropic Growth

**Scheme 1:**
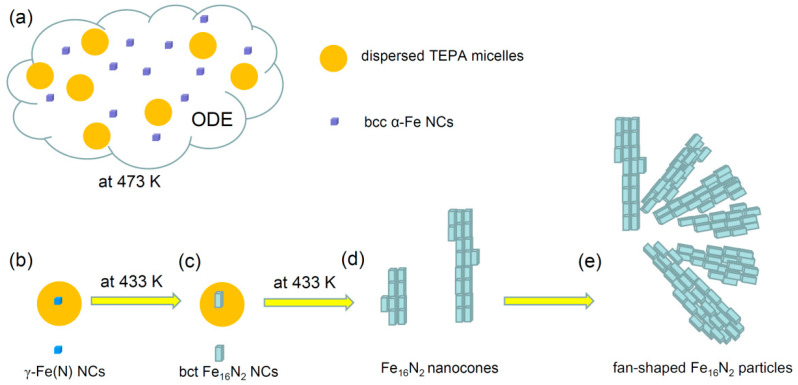
Schematic illustration of the growth mechanism of fan-shaped α″-Fe_16_N_2_@C nanocones in a nonpolar–polar ODE-TEPA microemulsion system. (**a**) Fe(CO)_5_ decomposed in the ODE to form bcc α-Fe NCs. (**b**) Fe NCs migrated into TEPA micelles and N atoms produced by decomposition of TEPA, which diffused into bcc Fe NCs to form Fe(N) NCs. (**c**) Formation of bct α″-Fe_16_N_2_ NCs. (**d**) α″-Fe_16_N_2_ NCs assembled to α″-Fe_16_N_2_ nanocones. The carbon shells formed on the surface of α″-Fe_16_N_2_ were omitted for clarity. (**e**) α″-Fe_16_N_2_@C nanocones agglomerated to form fan-shaped particles due to the magnetostatic interaction.

A previous study of the ε-Fe_3_N_1+x_@C nanoparticles revealed the nitridation and growth mechanism of core/shell structured ε-Fe_3_N_1+x_@C nanoparticles in a low-temperature wet chemical route [31]. In contrast to the nearly spherical shape of ε-Fe_3_N_1+x_@C nanoparticles, the anisotropic α″-Fe_16_N_2_ nanocones may possess a different growth mechanism. Scheme 1 presents the anisotropic growth mechanism of the α″-Fe_16_N_2_ nanocones in the ODE-TEPA solution reaction system. Unlike a previous oil-in-water (o/w) microemulsion that was stable below 333 K [36], a stable TEPA-in-ODE (TEPA/ODE) microemulsion was formed by simply heating a mixture of TEPA, ODE and OLA at a temperature higher than 393 K. It should be noted that the OLA served as a surfactant for dispersing the α-Fe nanocrystals, which is not necessary for the formation of a stable TEPA/ODE microemulsion. When a mixed solution of 2 mL of Fe(CO)_5_, 6 mL of ODE and 2 mL of OLA in a syringe was injected into the TEPA/ODE microemulsion at 473 K, body-centered cubic (bcc) α-Fe nanocrystals (NCs) were first formed in the ODE phase by thermal decomposition of Fe(CO)_5_. Under magnetic stirring, the bcc α-Fe NCs were transferred into the TEPA micelles. Based on the reaction mechanism [31], the α-Fe NCs catalytically decomposed TEPA to form N and C atoms. It should be noted that the reaction rate for the formation of C and N atoms may have been slow due to a reaction temperature of 433 K, which is much lower than the reaction temperature of 533 K for the ε-Fe_3_N_1+x_@C nanoparticles [31]. At the initial stage, a small amount of N atoms diffused into α-Fe NCs to form Fe(N) NCs. With increasing reaction time, more N atoms participated in the nitridation reaction and tetragonal α″-Fe_16_N_2_ NCs would be formed. In the case of synthesis of ε-Fe_3_N@C nanoparticles in TEPA, Fe NCs easily agglomerated to form nearly spherical nanoparticles with a wide particle size distribution of 100–500 nm in diameter [11]. However, in the present case, the interface between the nonpolar ODE phase and polar TEPA micelles controlled the Fe NCs transferring from the ODE phase to the TEPA phase. Once the tetragonal α″-Fe_16_N_2_ NCs were formed in the TEPA micelles, the magnetic α″-Fe_16_N_2_ NCs could assemble along the crystallographic c axis of α″-Fe_16_N_2_ NCs to form α″-Fe_16_N_2_ nanocones. Magnetocrystalline anisotropy energy is suggested as the driving force because the easy magnetization direction of tetragonal α″-Fe_16_N_2_ NCs was along the c axis. With the reaction going on, more and more C atoms were left on the surfaces of α″-Fe_16_N_2_ nanocones, resulting in a core/shell structure. The reaction would be cut off when the α″-Fe_16_N_2_ nanocones were completely covered by the carbon shell. The overall reaction processes can be speculated as following the reaction Equations (1)–(4):

Fe(CO)_5_ = Fe + 5CO (1)

2C_8_H_23_N_5_ = 10N + 16C +23H_2_ (2)

2N = N_2_ (3)

16Fe + 2N = Fe_16_N_2_ (4)

The catalytic decomposition of C_8_H_23_N_5_ (TEPA) should occur on the surface of α-Fe NCs due to the large surface energy. Smaller α-Fe NCs allowed easier production and diffusion of N atoms into them for the synthesis of the iron nitride phase [31]. When the reaction was stopped by cooling the reaction temperature from 433 K to room temperature, the TEPA/ODE microemulsion broke and the reaction system separated into a TEPA layer and an ODE layer at about 393 K. Meanwhile, the α-Fe NCs left in the ODE phase were magnetically attracted to the thinner end of the α″-Fe_16_N_2_@C nanocones due to higher magnetic flux density than that on the thicker end (see Figure 1d). The formation of fan structures may have been driven by the reduction of high surface energy and/or by the magnetostatic energy. However, the thinner ends of the α″-Fe_16_N_2_ nanocones directed the centers of the fan-shaped particles due to a higher magnetic flux density of the thinner end than the thicker end, indicating that magnetostatic energy was the dominant driving force in the formation of fan-shaped particles. The α-Fe NCs were oxidized to Fe_3_O_4_, while α″-Fe_16_N_2_ nanocones were protected by carbon shells from oxidation in an air atmosphere, in good agreement with the XPS and HRTEM results. As a result, anisotropic α″-Fe_16_N_2_@C nanocones with a core-shell structure were successfully synthesized in a new TEPA/ODE microemulsion system at a low temperature of 433 K and six days.

### 3.3. Thermal Property

**Figure 4:**
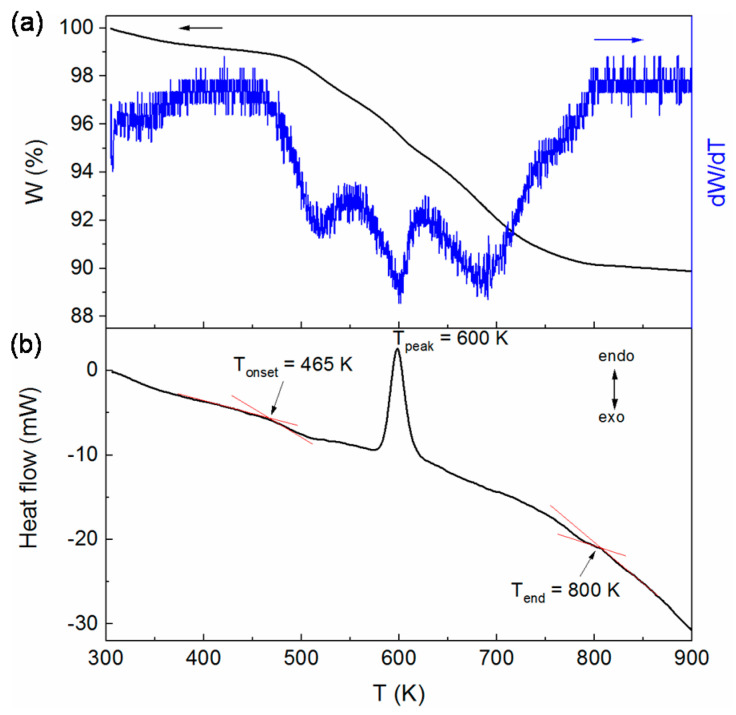
(**a**) TGA and dW/dT curves, (**b**) DSC curve of the α″-Fe_16_N_2_@C nanocones recorded in the heating process in a temperature range between 300 and 900 K.

**Figure 5:**
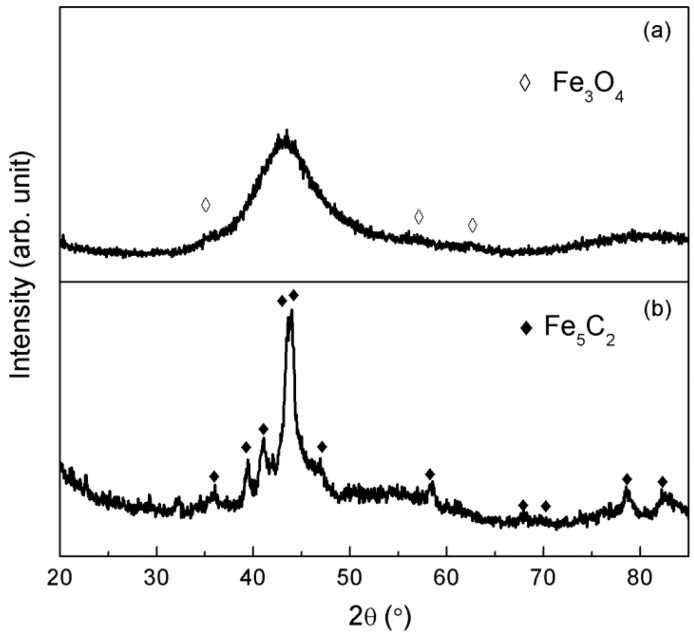
XRD patterns of the products heated at (**a**) 483 K and (**b**) 600 K in a vacuum.

Thermal analyses of the α″-Fe_16_N_2_@C nanocones synthesized at 433 K for six days are shown in Figure 4. The TGA curve in Figure 4a exhibits two steps of weight (*W*) loss in a temperature between 310 K and 800 K. The first step is a slight weight loss (~1 wt.%) below an onset temperature (*T*_onset_ = ~470 K) due to the loss of organic molecules adsorbed on the surface of nanocones. The *T*_onset_ corresponds to an onset temperature for the decomposition of α″-Fe_16_N_2_ due to high temperature. The second step covers a broad temperature range between *T*_onset_ and 800 K. The temperature dependence of d*W*/d*T* clearly displayed that the stage between 470 K and 600 K corresponded to release of the N atoms from α″-Fe_16_N_2_, while the stage between 600 K and 800 K may have originated from the reduction of iron oxide by the carbon shells of α″-Fe_16_N_2_@C nanocones following a possible reaction of Fe_3_O_4_ + 4C = 3Fe + 4CO. As a result, the weight loss of ~8 wt.% in the second step should be ascribed to the losses of ~3 wt.% nitrogen atoms from α″-Fe_16_N_2_ and ~5 wt.% oxygen/carbon atoms due to the losses of oxygen atoms and carbon atoms. Our previous work [37] indicates that amorphous iron oxides on the surfaces of ε-Fe_3_N nanoparticles become Fe_3_O_4_ and Fe_2_O_3_ crystals at a temperature near 623 K, but loss of oxygen at a temperature between 623 and 793 K. This suggests that the α″-Fe_16_N_2_@C nanocones were metastable below ~470 K and the weight loss of ~3 wt.% from 470 to 600 K is in good agreement with the weight percentage of N atoms in α″-Fe_16_N_2_. Besides the reactions for the decomposition of α″-Fe_16_N_2_ and the redox reduction of Fe_3_O_4_, an endothermic reaction also happened at about 600 K, as revealed by the DSC curve (Figure 4b). The possible reaction was 5Fe_16_N_2_ + 32C = 16Fe_5_C_2_ + 5N_2_. Compared with Figure 1b, the XRD pattern in Figure 5a reveals almost no change of the α″-Fe_16_N_2_@C nanocones heat-treated at 483 K. However, the main phase became Fe_5_C_2_ (JCPDS card no. 51-0529) when the α″-Fe_16_N_2_@C nanocones were heated to 600 K (Figure 5b). The main phase of Fe_5_C_2_ heated at 600 K proves the loss of N atoms from the α″-Fe_16_N_2_@C nanocones at a temperature lower than 600 K.

### 3.4. Magnetic Properties

**Figure 6:**
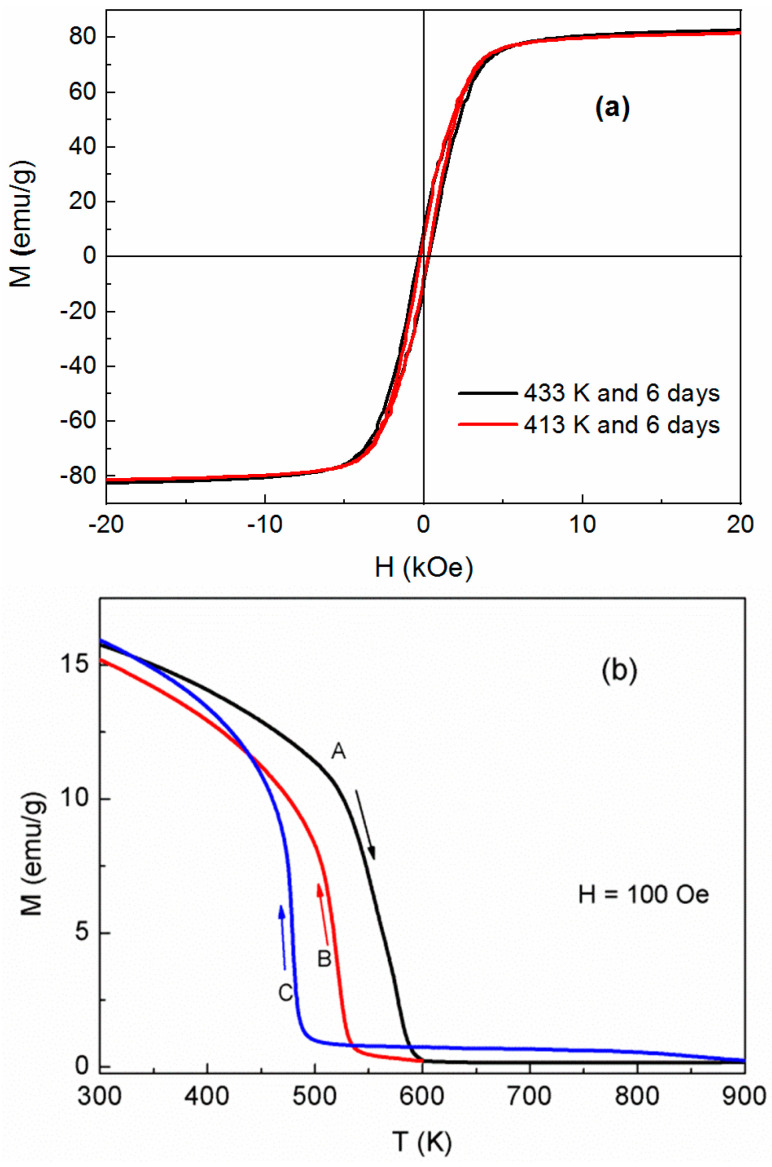
(**a**) Magnetic hysteresis loops of the α″-Fe_16_N_2_@C nanocones synthesized at 433 K for six days and at 413 K for six days, respectively. (**b**) Temperature dependence of magnetization of the α″-Fe_16_N_2_@C nanocones synthesized at 433 K and six days in (A) a warming process between 300 and 900 K and the cooling processes from different temperature of (B) 600 K and (C) 900 K, respectively, to 300 K.

Figure 6a represents the room-temperature magnetic hysteresis loops of the α″-Fe_16_N_2_@C nanocones synthesized at 433 K for six days and at 413 K for six days, respectively, with characteristics of ferromagnetic materials. The saturation magnetization (*M_S_*) and the coercivity (*H_C_*) were respectively 83 emu/g and 320 Oe at 300 K for the α″-Fe_16_N_2_@C nanocones synthesized at 433 K for six days, while the *M_S_* and the *H_C_* became 81.6 emu/g and 250 Oe at 300 K for the α″-Fe_16_N_2_@C nanocones synthesized at 413 K for six days. The decrease in the magnetic properties of the α″-Fe_16_N_2_@C nanocones may be ascribed to a slight composition change of α″-Fe_16_N_2_ due to the reduced reaction temperature. Nevertheless, the saturation magnetizations were much lower than the previously reported *M_S_* (290 emu/g) for α″-Fe_16_N_2_ thin film [9]. According to our previous work on the carbon-coated ε-Fe_3_N_0.88_ nanoparticles, the carbon shells reduce the *M_S_* of ε-Fe_3_N_0.88_ nanoparticles by a value of 11.8% [11]. Thus, the *M_S_* value of the α″-Fe_16_N_2_ nanocones would slightly increase if removing the negative effect of diamagnetic carbon. Considering the XRD pattern in Figure 2c, the fraction of Fe_3_O_4_ nanoparticles was very small and can be neglected, while the minimum fraction of carbon was ~8 wt.% estimated based on the Fe_5_C_2_ phase in Figure 5b. Taking the diamagnetic susceptibility of about −1.4 × 10^−^^5^ emu/g · Oe at 300 K for carbon [38], the *M_S_* of α″-Fe_16_N_2_ nanocones increased at least to 90.24 emu/g at 300 K by removing the C shells from the α″-Fe_16_N_2_@C nanocones. Figure 6b plots the temperature dependence of magnetization (*M-T*) of the α″-Fe_16_N_2_@C nanocones in the warming and the cooling processes in a temperature range between 300 and 900 K. The *M-T* curves in the warming and the cooling processes were irreversible. The *M-T* curve in the warming process A shows a decrease of magnetization with an increasing temperature with a knee point at about 510 K. It reveals the thermal decomposition of α″-Fe_16_N_2_, which was a little higher than the temperature determined by thermal analysis (Figure 4). Below the thermal decomposition temperature of 510 K, the α″-Fe_16_N_2_ was metastable (Figure 5a). Above 600 K, the magnetization in the warming process became almost a constant. The *M-T* curve recorded in the cooling process B (Figure 6b) presents a magnetic transition temperature at 528 K, which was determined by the point of intersection of the two tangents around the inflection point of the cooling *M-T* curve. The ferromagnetic–paramagnetic transition at 528 K should be ascribed to the Fe_5_C_2_ phase determined by the XRD pattern (Figure 5b). Such a *T_C_* of 528 K for Fe_5_C_2_ agrees with the *T_C_* value previously reported for Fe_5_C_2_ [39]. There were no magnetic transitions in the cooling process B between 550 and 600 K and in the warming process A between 600 and 900 K. The absence of magnetic phases of ε-Fe_3_N (*T_C_* = 575 K), γ-Fe_4_N (*T_C_* = 760 K) and Fe_3_O_4_ (*T_C_* = 864 K) reveals that all the N atoms had been released from the α″-Fe_16_N_2_ nanocones and Fe_3_O_4_ had been reduced by carbon in the warming process A. Figure 4a suggests that the reduction of Fe_3_O_4_ by carbon happened at a temperature range of 600–800 K, revealing a mass loss about 5 wt.% due to the loss of oxygen. The magnetic product of Fe_3_O_4_ reduced by carbon is α-Fe. The *M-T* curve recorded in the cooling process C (Figure 6b) shows two different magnetic transitions at 483 and above 900 K, respectively. The magnetic transition temperature of 483 K is in good agreement with the *T_C_* of Fe_3_C, while the magnetic transition above 900 K may be ascribed to α-Fe. However, the change of the magnetization after raising the temperature from 700 to 900 K in process C was not obvious due to a small amount of α-Fe and a higher magnetic transition temperature of α-Fe (*T_C_* ≈1043 K). It is reasonable that the magnetizations in the process B at the temperature range of 500–600 K (Figure 6b) were smaller than those in the process A due to existence of α″-Fe_16_N_2_ and in process C due to existence of a small amount of Fe. It is possible that the α-Fe partially diffused into the Fe_5_C_2_ to form Fe_3_C following the reaction equation of Fe_5_C_2_ + Fe = 2Fe_3_C, but leaving a small amount of α-Fe in the product heated up to 900 K.

Despite the low saturation magnetization and coercivity at room temperature, anisotropic 1D α″-Fe_16_N_2_@C nanocones were synthesized for the first time in a one-pot chemical solution method. It has been found that diamagnetic carbon shells weaken the saturation magnetization of α″-Fe_16_N_2_ nanocones although the carbon shells can protect the α″-Fe_16_N_2_@C nanocones from oxidation in an air atmosphere. The Fe NCs with small particle sizes were easily nitridized to form α″-Fe_16_N_2_, but small grain sizes of α″-Fe_16_N_2_ NCs may be detrimental to their high coercivity performance. The theoretical shape anisotropy field was 2 *πM_S_* for the nanowire with very high aspect ratio. Moreover, in nanowire arrays, the dipolar interaction between wires becomes important. The maximum field originating from dipole–dipole interactions was −2π*PM_S_* when the magnetization was saturated perpendicular to the wire axis, and 4*πPM_S_* when the magnetization was saturated along the wire axis, where *P* was a packing density of the nanowire array [40]. The low coercivity at room temperature for the present anisotropic 1D α″-Fe_16_N_2_@C nanocones may have resulted from the fan-like agglomerated structure, which can form a magnetic vortex different from the magnetic state produced by separate elongated particles. Therefore, to enhance the hard magnetic properties of the α″-Fe_16_N_2_@C nanocones, it is worth studying the syntheses of α″-Fe_16_N_2_ nanocones/nanorods with controllable grain/particle sizes and alignments of the separate 1D nanorods/nanocones instead of a fan-like structure. Moreover, as has been demonstrated in the ε-Fe_3_N_1+x_ (*x* ≤ 0) [11], finely engineering the N content in the iron nitride nanostructures and removing the impurity are also critical to increase the saturation magnetization of α″-Fe_16_N_2_@C material. Our low-temperature chemical synthesis for 1D α″-Fe_16_N_2_ nanostructures has potential to obtain high permanent magnetic performance. Efforts to synthesis a magnetically oriented 1D α″-Fe_16_N_2_ hard magnet are still in progress.

## 4. Conclusions

We developed a facile protocol for synthesizing carbon-coated tetragonal α″-Fe_16_N_2_ nanocones by a new one-pot microemulsion method with Fe(CO)_5_ serving as the iron source and TEPA as the N/C source. Anisotropic growth of the tetragonal α″-Fe_16_N_2_@C nanocones is ascribed to the strong magnetocrystalline anisotropy energy of α″-Fe_16_N_2_ nanocrystals. α″-Fe_16_N_2_@C nanocones agglomerated to form a fan-shaped microstructure due to the magnetostatic energy. 1D α″-Fe_16_N_2_@C nanocones have excellent oxidation resistance. Despite their hard magnetic characteristics with room-temperature saturation magnetization and coercivity of 82.6 emu/g and 320 Oe, respectively, the energy produced is low due to the low saturation magnetization and coercivity of the material. However, as has been demonstrated, the magnetic properties are easily tunable by the content of ordering N atoms and grain sizes. Systematic experimental work is under way to enhance the saturation magnetization and coercivity of 1D α″-Fe_16_N_2_@C nanocones. If these properties are enhanced, 1D α″-Fe_16_N_2_ aligned hard-magnetic materials can be a low-cost, nontoxic alternative to noble-metal- or rare-earth-element-based advanced magnets.

## Data Availability

The data is available on reasonable request from the corresponding author.
